# Genome-Scale Metabolic Model of Infection with SARS-CoV-2 Mutants Confirms Guanylate Kinase as Robust Potential Antiviral Target

**DOI:** 10.3390/genes12060796

**Published:** 2021-05-24

**Authors:** Alina Renz, Lina Widerspick, Andreas Dräger

**Affiliations:** 1 Department of Computer Science, University of Tübingen, 72076 Tübingen, Germany; renz@informatik.uni-tuebingen.de; 2 Cluster of Excellence ‘Controlling Microbes to Fight Infections’, University of Tübingen, 72076 Tübingen, Germany; 3 Computational Systems Biology of Infections and Antimicrobial-Resistant Pathogens, Institute for Bioinformatics and Medical Informatics (IBMI), University of Tübingen, 72076 Tübingen, Germany; 4 Bernhard Nocht Institute for Tropical Medicine, Virus Immunology, 20359 Hamburg, Germany; lina.widerspick@bnitm.de; 5 German Center for Infection Research (DZIF), Partner Site Tübingen, 72076 Tübingen, Germany

**Keywords:** SARS-CoV-2, COVID-19, flux balance analysis (FBA), genome-scale metabolic models, target identification, reaction knock-out, structural proteins, purine metabolism, pyrimidine metabolism, B.1.1.7, B.1.351, B.1.617, B.1.28, B.1.427/B.1.429

## Abstract

The current SARS-CoV-2 pandemic is still threatening humankind. Despite first successes in vaccine development and approval, no antiviral treatment is available for COVID-19 patients. The success is further tarnished by the emergence and spreading of mutation variants of SARS-CoV-2, for which some vaccines have lower efficacy. This highlights the urgent need for antiviral therapies even more. This article describes how the genome-scale metabolic model (GEM) of the host-virus interaction of human alveolar macrophages and SARS-CoV-2 was refined by incorporating the latest information about the virus’s structural proteins and the mutant variants B.1.1.7, B.1.351, B.1.28, B.1.427/B.1.429, and B.1.617. We confirmed the initially identified guanylate kinase as a potential antiviral target with this refined model and identified further potential targets from the purine and pyrimidine metabolism. The model was further extended by incorporating the virus’ lipid requirements. This opened new perspectives for potential antiviral targets in the altered lipid metabolism. Especially the phosphatidylcholine biosynthesis seems to play a pivotal role in viral replication. The guanylate kinase is even a robust target in all investigated mutation variants currently spreading worldwide. These new insights can guide laboratory experiments for the validation of identified potential antiviral targets. Only the combination of vaccines and antiviral therapies will effectively defeat this ongoing pandemic.

## 1. Introduction

Since its emergence in December 2019 [1], individual cases of Severe Acute Respiratory Syndrome (SARS) coronavirus (CoV) type 2 (SARS-CoV-2) infections have evolved into an uncontrolled pandemic. As a result, more than 2.8 million people have lost their lives to or with Coronavirus Disease 19 (COVID-19) by March 2021. COVID-19 symptoms range from pneumonia to severe lung, heart, liver, kidney, neurological or gastrointestinal dysfunction [2]. While great efforts have been employed to provide effective SARS-CoV-2 vaccines [3,4], their success is overshadowed by the emergence of viral escape mutants and the shortcomings in developing targeted antiviral treatments. A meta-analysis by [5] demonstrates that in non-severe cases of COVID-19, there is little to no evidence for effective use of ribavirin, hydroxychloroquine, umifenovir, lopinavir/ritonavir, or interferon [5]. Even the putative effectiveness of remdesivir is questionable [6,7].

While antiviral medication development was less fruitful, as of March 2021, there are 13 vaccines for SARS-CoV-2 in use, most of them targeting the spike (S) protein [3,8]. Albeit the successes in vaccine development, reports of mutations are increasing. Some of these mutations are even bypassing the immunity provided by several vaccine candidates. Five mutation variants have prevailed, disseminate rapidly, and are classified as variants of concern or variants of interest: (i) B.1.1.7, first detected in the United Kingdom; (ii) P.1 (also called B.1.1.28), first detected in Japan and Brazil; (iii) B.1.351, first detected in South Africa; (iv) B.1.427/B.1.429, first detected in the US [9,10]; and (v) B.1.617, first detected in India [11]. While the consequences of some of these mutations for vaccine efficacy have been reported, the metabolic implications of them remain unclear.

SARS-CoV-2 is a member of the *Betacoronavirus* genus within the *Nidovirales* order [4,12]. The virus has a 27 kb to 32 kb positive sense, single-stranded RNA genome encoding 26 proteins, including the four structural proteins spike (S), envelope (E), membrane (M) and nucleocapsid (N) [4,13]. The S trimers [14,15] scan the host cells surface for the viral entry receptor angiotensin converting enzyme 2 (ACE-2) and therefore initiate the entry process [1,4]. The structural proteins E and M facilitate viral transport, assembly, budding, and release of SARS-CoV-2 virions from infected host cells [1,4]. While N is expressed within the host cytoplasm, the other structural proteins S, E, and M are translated within the endoplasmic reticulum–Golgi intermediate compartment (ERGIC) of the host cell [2,4]. SARS-CoV-2 N supports replicating the viral genome in the cytoplasm and encloses novel viral RNA to form viral ribonucleoprotein complexes (vRNPs) [2]. During the viral replication process’s final steps, these cytoplasmic vRNPs are assembled with S, E, and M proteins within the ERGIC [2,4]. The mature virions bud at the ERGIC membrane, forming vesicles which are subsequently released from the host cell via exocytosis [2,4].

Viral lipid envelopes protect the vRNPs and facilitate the particles’ entry into host cells [16]. They are usually acquired via budding from the plasma membrane or other cellular organelles [16,17]. Viruses specifically modify host membrane structures, the composition, and the whole host lipid metabolism to favor viral replication [16,18,19]. Many viruses exploit spatiotemporally enriched microdomains or rafts containing different lipid species [19]. To this end, cholesterol, for instance, increases host membrane fluidity for efficient viral entry, replication, and budding, while phosphatidylserine supports viral entry [19]. Altogether, various modifications in viral egress areas determine the differing composition of viral envelopes, thereby influencing their stability and infectivity [19]. As SARS-CoV-2 buds from the ERGIC [2,4], its envelope lipid bilayer resembles this host organelle’s composition [2,4]. The viral membrane formation mostly requires cholesterol and phospholipids, while sphingomyelin and cardiolipin are presumably less abundant [20,21].

In our previous work, we have generated an integrated human-virus metabolic model, which combines flux balance analysis (FBA) and flux variability analysis (FVA) to model the metabolic changes within SARS-CoV-2 infected human alveolar macrophages [22]. The GEM is based on the already published and well-developed human alveolar macrophage model iAB-AMØ-1410 by by Bordbar et al. [23]. Disabling viral replication in human alveolar macrophages might be an early way of intervention and prevention of the virus’s further spread. The model was employed to predict putative antiviral targets such as guanylate kinase 1 (GK1) or the availability of l-isoleucine and l-lysine [22]. Some of these potential targets may be directly targeted by small molecules or antivirals [24,25,26]. Increasing knowledge of SARS-CoV-2 facilitates the model’s improvement by incorporating recent findings of the copy number of the structural proteins [22,27]. The stoichiometric coefficients of the metabolic requirements for amino acids and nucleotides and energy requirements can be refined to predict the viral replication capacity better. Additionally, the lipid requirements were now accounted for in the viral biomass objective function (VBOF). This study presents an updated version of the integrated alveolar macrophage SARS-CoV-2 GEM and the consequences of prominent mutations for predicted metabolic targets.

## 2. Materials and Methods

### 2.1. Correcting the Copy Number of Structural Proteins

In the previous version of the VBOF, the copy number of structural proteins was not yet known. We conducted extensive literature research to identify the precise copy number of each structural protein individually. The search was mainly focused on SARS-CoV-2 directly. However, if no information was found for the novel coronavirus, we also searched for information on closely related coronaviruses.

With the identified copy numbers (see Table 2), the stoichiometric coefficients of the nucleotides, amino acids, and energy requirements were re-calculated, as Renz et al. state [22]. However, instead of using a general copy number for all structural proteins, as Aller et al. describe [27], the individual copy numbers of the respective structural proteins were used.

After the VBOF was updated with the corrected stoichiometric coefficients, the knock-out and host-derived enforcement analyses were repeated, as Renz et al. describe [22]. The knock-out experiments were performed by subsequently knocking out each reaction and evaluating its effect on the host’s maintenance and viral replication capacity (VBOF). For the host-derived enforcement analyses, the FVA was used to determine flux ranges that allow for 100% maintenance of the host, while decreasing the viral growth by at least 20%. The adapted host-derived enforcement algorithm was used, as Renz et al. describe [22].

### 2.2. Testing the Targets’ Robustness against for Several Mutations

The Global Initiative on Sharing All Influenza Data (GISAID) database has a collection of more than 1.5 million viral sequences of SARS-CoV-2 (May 2021). We set the following filters for the sequences: (i) variant (VUI202012/01 GRY (B.1.1.7) for variant B.1.1.7; GH/501Y.v2 (B.1.351) for variant B.1.351; GR/501Y.V3 (P.1) for variant B.1.1.28; GH/452R.V1 (B.1.429+B.1.427) for variants B.1.429 and B.1.427; and G/452R.V3 (B.1.617+) for variant B.1.617) and (ii) location (Europe/United Kingdom for variant B.1.1.7; Africa for variant B.1.351; South America for variant B.1.1.28; North America/USA for variants B.1.429 and B.1.427; and Asia/India for variant B.1.617). We randomly downloaded ten sequences from each mutation variant with the filters set as described. In addition to the sequences, we downloaded the mutation information given in the metadata. All tested mutations are listed in the Supplementary Table S1. With this information, the stoichiometric coefficients for the VBOF were calculated for every downloaded mutation. As the calculation of the nulceotides’ stoichiometric coefficients requires the nulceotide sequence, the downloaded sequences were used directly for this step. For the calculation of the amino acids’ coefficients, we used the annotated protein sequence of the SARS-CoV-2 reference sequence (NCBI accession: NC_045512.2) and the mutation information extracted from the metadata files. An algorithm adapted the amino acids from the protein sequence in accordance with the defined mutations, including substitutions, deletions, and introductions of stop codons. With the calculation of the energy requirements and pyrophosphate liberation, all stoichiometric coefficients for the VBOF were available and could be compared. For the first comparison, the mean and standard deviation of all mutations was calculated for each coefficient. These mean values were compared to the wildtype stoichiometric coefficients by calculation the difference. In subsequent analysis, the mean was calculated for the five mutation variants and was then compared to the wildtype. Again, the difference between the coefficients was calculated and visualized. With all generated VBOFs, the reaction knock-out experiments were repeated, as described in the previous section.

### 2.3. Lipids as Part of the Viral Biomass Objective Function

**Figure 1:**
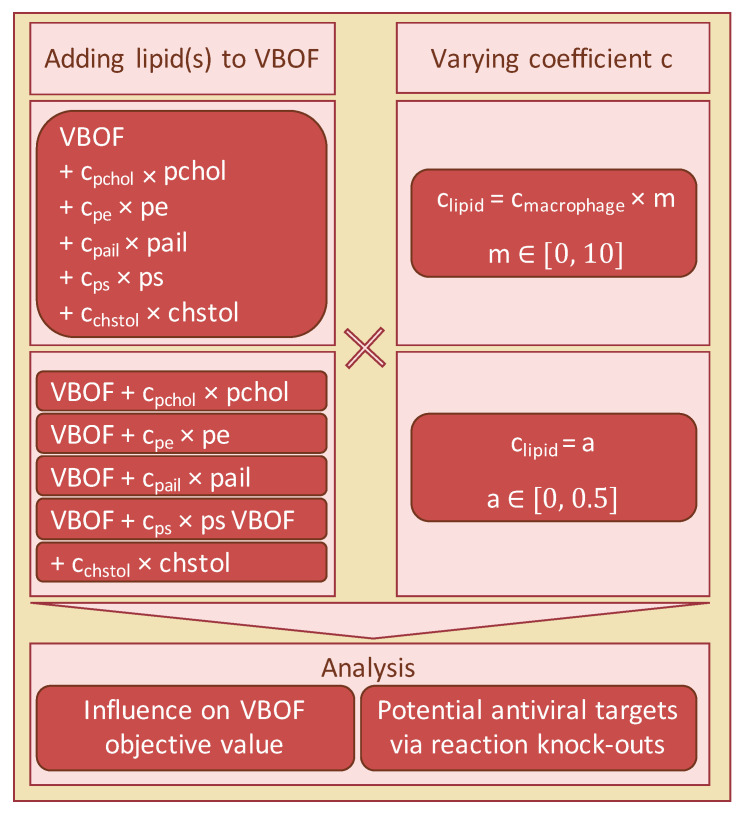
Workflow for the investigation of lipids’ influence on the VBOF. The five lipids phosphatidylcholine (pchol), phosphatidylethanolamine (pe), phosphatidylinositol (pail), phosphatidylserine (ps), and cholesterol (chstol) were added together and individually to the VBOF. The stoichiometric coefficients were either an absolute value identical for all lipids, or the initial stoichiometric coefficient from the macrophage biomass function factorized with a multiplication-coefficient. For all scenarios, the influence of the different VBOFs on the objective value was analyzed. Additionally, potential antiviral targets were examined using reaction knock-outs.

Literature research was conducted to identify potential fatty acids that occur in the capsid of SARS-CoV-2. As no lipidomics data of SARS-CoV-2 existed at the time of writing, we focused on the five identified lipids phosphatidylcholine, phosphatidylethanolamine, phosphatidylinositol, phosphatidylserine, and cholesterol. The influence of the individual lipids’ inclusion into the VBOF on the objective value when optimizing for the VBOF was evaluated. An overview of the overall procedure for testing the lipids’ influence is given in Figure 1.

As no data were available for the amount of the respective lipids in one virion, we varied the stoichiometric coefficients between 0 and 0.5. The stoichiometric coefficients of the lipids within the macrophage’s biomass maintenance function varied from 0.00102 for phosphatidylserine to 0.0315 for phosphatidylcholine (see also Table 1).

With the variation of the stoichiometric coefficients between 0 and 0.5, we covered the 14 to 490-fold increase of the stoichiometric coefficients, depending on their initial value. In the next step, all lipids were added simultaneously to the VBOF. We evaluated the VBOF’s objective value using both the lipids’ stoichiometric coefficients from the macrophage’s maintenance function and their ten-fold value.

To evaluate the effect of the lipids’ inclusion on the potential antiviral targets, we again used the stoichiometric coefficients of the macrophage’s maintenance function and a multiplication coefficient, ranging from 0 to 10 as the actual coefficient of the lipids is unknown. We conducted the knock-out experiments as Renz et al. describe [22] for each tested coefficient by knocking out each reaction individually and analyzing its effect on both the viral growth and the host’s maintenance function. While varying the multiplication coefficient, two additional reactions occurred, whose knock-out decreased the viral growth rate.

To investigate, which lipid influences the knock-out experiments most, we again analyzed the lipids individually. As done for the effect on the VBOF’s objective value, we first varied the stoichiometric coefficients between 0 and 0.5. Subsequently, we used a multiplication coefficient ranging from 0 to 10, which was multiplied with the coefficient of the macrophage’s maintenance function (see Table 1).

## 3. Results

### 3.1. Correcting the Copy Number of Structural Proteins

The single-stranded RNA genome of SARS-CoV-2 has 26 proteins [13], including four structural proteins. These four structural proteins need to be produced by the host in higher amounts than the non-structural proteins. However, the actual number of copies of each structural protein was unknown when the novel coronavirus arose, and the first studies were conducted at the beginning of the year 2020.

After extensive literature research, we collected the latest information about the copy number of the structural proteins of SARS-CoV-2. [15] identify on average 40 copies of the trimeric spike (S) protein on the surface of SARS-CoV-2, resulting in 120 copies of the S protein. [14] estimate the number of S trimers per virion to be 48, resulting in a similar copy number range as [15]. Since [15] use in situ structural analysis and [14] use mathematical estimations, we chose to use a copy number of 120 S proteins for further analysis (see Table 2). The number of the envelope (E) proteins is approximated to 20 copies [29] based on analyses of the OC43 human coronavirus (hCOV) [30] and the transmissible gastroenteritis virus (TGEV) [31]. Exactly like SARS-CoV-2, both viruses belong to the family of *Coronaviridae*, and hCOV also belongs to the same genus *Betacoronavirus* as SARS-CoV-2. Currently, no numbers for the E protein are available for SARS-CoV-2. For that reason, the number is approximated from related coronaviruses. The nucleocapsid (N) packs the viral RNA in so-called vRNPs. [14] observe 38 vRNPs per SARS-CoV-2 virion [14]. Approximately 12 copies of the N protein are located in one vRNP in SARS-CoV-2 [32,33]. Multiplying those two numbers results in 456 copies of the N protein. The amount of membrane proteins is not yet determined for SARS-CoV-2. [13] provide key numbers about SARS-CoV-2, including the copy numbers of the S, M, N, and E protein. However, all copy numbers are derived from SARS-CoV-1 or TGEV. We found precise numbers for the copy number of N proteins in SARS-CoV-2, and [34] determine the estimated ratios of M to N proteins ranging from 3M:1N to 1M:1N with 730 to 2200 N proteins per virion [34]. With this information at hand, we estimated the copy number of M proteins to 1000 by doubling the number of N proteins and rounding them up. The ratio of 2M:1N was chosen based on the article of [13], where the number of N proteins is stated as 1000 copies for SARS-CoV-1 and the number of M proteins as 2000. All used copy numbers are listed in Table 2.

With the updated copy numbers, the stoichiometric coefficients of the nucleotides, amino acids, and energy requirements were re-calculated for the viral biomass objective function (VBOF) of SARS-CoV-2. The subsequent analyses for identifying potential antiviral targets consisted of knock-out and host-derived enforcement experiments, as Renz et al. describe [22]. The guanylate kinase 1 (GK1) remains a promising antiviral target after the adaptions of the copy number of structural proteins based on the knock-out experiments.

**Figure 2:**
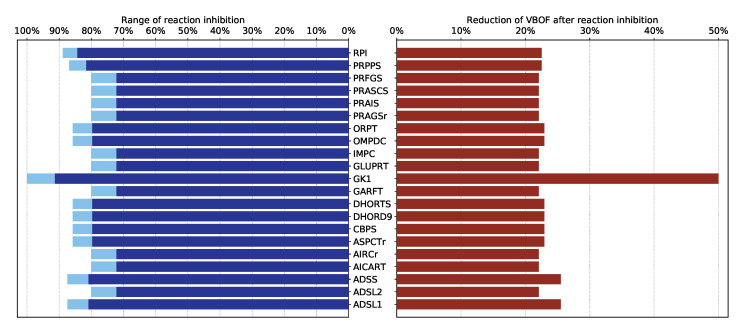
Results of the host-derived enforcement experiments. With the help of the host-derived enforcement, the range and effect of reaction inhibitions on the VBOF can be investigated while keeping the host’s maintenance at 100%. The minimum possible reaction inhibition rate to reduce the viral replication capacity (VBOF) is given in dark blue. The maximum inhibition of the reaction does not harm the host’s maintenance and is indicated in light blue. The reduction of the VBOF is given in comparison to the un-inhibited state. All reaction identifiers are BiGG identifiers [28]. Table A1 lists all reaction identifiers with their corresponding reaction name and the subsystem they occur in.

The results of the host-derived enforcement analyses were dependent on the Copy number of structural proteins [22]. As we now identified more precise copy numbers, we can also determine the host-derived enforcement analysis results more precisely. In total, 21 reactions were identified, whose inhibition decreases the viral replication capacity by at least 20% without harming the host’s maintenance (100%). These reactions, their inhibition range, and the reduction of the VBOF are visualized in Figure 2. Reactions could be inhibited between 72% and 89%. As seen in the knock-out experiments, the guanylate kinase 1 (GK1) is the only reaction where a complete inhibition (100%) is possible. The ribose-5-phosphate isomerase (RPI) and phosphoribosylpyrophosphate synthetase (PRPPS) are part of the pentose phosphate pathway. glutamine phosphoribosyldiphosphate amidotransferase (GLUPRT), phosphoribosylglycinamide synthase (PRAGSr), phosphoribosylglycinamide formyltransferase (GARFT), phosphoribosylformylglycinamidine synthase (PRFGS), phosphoribosylaminoimidazole synthase (PRAIS), Phosphoribosylaminoimidazole carboxylase (AIRCr), phosphoribosylaminoimidazolesuccinocarboxamide synthase (PRASCS), phosphoribosylaminoimidazolecarboxamide formyltransferase (AICART), and inosine monophosphate (IMP) cyclohydrolase (IMPC) are involved in the purines’ biosynthetic pathway, more precisely in the biosynthesis of IMP [35]. Reactions associated with the purine adenosine monophospate (AMP) biosynthesis were also identified as potential targets, namely adenylosuccinate synthase (ADSS), and adenylosuccinate lyase 1 and 2 (ADSL1, ADSL2) [35].

Besides the reactions associated with the purine metabolism, the host-derived enforcement analysis also reported reactions from the pyrimidine biosynthesis, such as the carbamoyl-phosphate synthase (CBPS), aspartate carbamoyltransferase (ASPCTr), dihydroorotase (DHORTS), dihydoorotic acid dehydrogenase (DHORD9), orotate phosphoribosyltransferase (ORPT), and orotidine-5’-phosphate decarboxylase (OMPDC) [36].

### 3.2. Testing the Targets’ Robustness for Several Mutations

#### 3.2.1. Analysis of Mutant-Specific Variations in the Viral Biomass

Novel mutations of SARS-CoV-2 emerge on a daily basis. Five mutation variants have prevailed, disseminate rapidly, and are classified as variants of concern or variants of interest: (i) B.1.1.7, (ii) P.1 (also called B.1.1.28), (iii) B.1.351, (iv) B.1.427/B.1.429 [9,10], and (v) B.1.617 [11]. The GISAID was launched in 2008 to promote the international sharing of virus data [37,38]. When the novel coronavirus emerged, GISAID was expanded by a database for sharing sequenced viral genomes of SARS-CoV-2 globally. At the time of writing, more than 1.5 million viral sequences of SARS-CoV-2 are collected in the database. To investigate the mutations’ effect on the previously identified potential antiviral targets, sequences of each mutation variant were downloaded from GISAID and analyzed. The stoichiometric coefficients of each variant were calculated as Renz et al. describe [22]: For the calculation of the nucleotides’ stoichiometric coefficients, the downloaded RNA sequence was used. The amino acids’ stoichiometric coefficients were calculated using the provided information about the identified mutations and the reference (wildtype) protein sequence of the first sequenced SARS-CoV-2. With this information, the abundance of the different amino acids in the different proteins was adapted for each mutation variant. The nucleotide and amino acid counts were subsequently used to calculate the pyrophosphate liberation and the adenosine triphosphates (ATPs) requirements. For each downloaded mutation variant, an individualized VBOF was created with the calculated stoichiometric coefficients.

**Figure 3:**
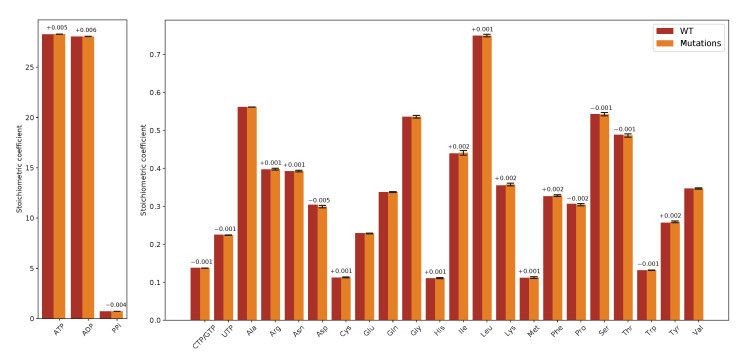
Difference of stoichiometric coefficients between wildtype (WT) and all mutations. The stoichiometric coefficients for all metabolites participating in the viral biomass objective function (VBOF) are compared. WT stoichiometric coefficients are indicated in red, the mean stoichiometric coefficients of all mutation variants are indicated in orange, including standard deviations (black). If the difference of the stoichiometric coefficients between WT and mutation variants was more than 0.001, the difference is indicated above the bars. The stoichiometric coefficients for the metabolites ATP, ADP and PPi, are higher compared to the other coefficients. The mutation variants’ mean coefficients show little deviation. Additionally, the differences between the stoichiometric coefficients of WT and mutation variants are very small.

To assess the mutations’ effect on the VBOF’s stoichiometric coefficients, we first calculated the mean and standard deviation from all stoichiometric coefficients for all mutations and compared them to the wildtype (WT) coefficients. The mean stoichiometric coefficients of the mutations are very similar to the wildtpye’s stoichiometric coefficents. The largest difference is observed for the amino acid l-aspartate: The stoichiometric coefficient for l-aspartate is decreased by on average 0.005 in the mutations compared to the wildtype. Figure 3 visualizes the comparison of the mutations’ mean stoichiometric coefficients with the wildtype coefficients.

**Figure 4:**
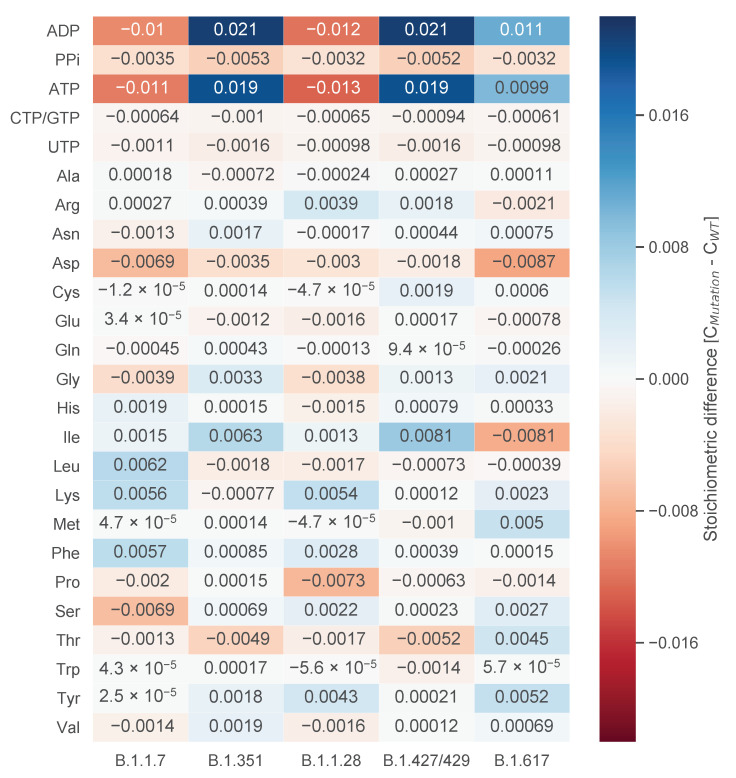
Difference of stoichiometric coefficients between wildtype (WT) and the individual mutations. The deviation between WT and the mean of the individual mutation variants was calculated. Higher stoichiometric coefficients in the mutation compared to the WT are indicated in blue, while lower stoichiometric coefficients are indicated in red. Based on similar sequence length for the mutation variants B.1.1.7 and B.1.1.28 and resulting similar total viral molar masses, a pattern emerges, which is most apparent for the stoichiometric coefficients of ATP and ADP. This pattern, however, is not present for all stoichiometric coefficients. The coefficient for l-serine, for example, is only decreased in the mutation variant B.1.1.7 based on two mutations in two structural proteins. Overall, the deviations from the WT are very small.

Since we analyzed five distinct mutation variants, the differences in the stoichiometric coefficients were examined further based on these variants. The mean for each stoichiometric coefficient was calculated variant-wise. With this mean, the deviation from the wildtype coefficient was calculated and visualized as a heat-map in Figure 4. This analysis gives further insight into the properties of the individual mutations.

One can observe a pattern for the stoichiometric coefficients of adenosine diphosphate (ADP) and ATP: While the mutation variants B.1.1.7 and B.1.1.28 have decreased stoichiometric coefficients (−0.01) compared to the wildtype, the variants B.1.351 and B.1.427/429 have increased stoichiometric coefficients (0.019 to 0.021). This pattern is most apparent for ADP and ATP, but can also be observed for other stoichiometric coefficients, such as for diphosphate (PPi), l-lysine, l-threonine, or l-valine. To further investigate this pattern, we examined the calculation for the stoichiometric coefficients. Each coefficient is set in relation to the total viral molar mass (Mv), which is the sum of the total molar mass of all nucleotides (Gi) and amino acids (Gj). The mutation variants B.1.1.7 and B.1.1.28 have a higher total viral molar mass compared to the mutation variants B.1.351 and B.1.427/429. This increased total viral molar mass is based on an increased molar mass of both nucleotides (Gi) and amino acids (Gj). As the stoichiometric coefficients for ADP and ATP larger than the other coefficients, this pattern is more apparent.

However, this pattern does not emerge in all stoichiometric coefficients. There are deviations for, e.g., l-serine. Only the mutation variant B.1.1.7 shows a decreased stoichiometric coefficient compared to the wildtype. We analyzed the documented mutations for this variant and identified two mutations in structural proteins, Spike S982A and N S235F, which only occur in this variant. In both cases, the amino acid l-serine is substituted by another amino acid. As both mutations occur in structural proteins with copy numbers of 120 and 456, respectively, their influence on the amount of amino acid and, thus, the stoichiometric coefficient, is noticeable. Compared to the other mutation variants, variant B.1.1.28 has the highest increase in the stoichiometric coefficient for l-serine. This could be explained by two mutations specific for this variant in the structural spike protein: Spike P26S and Spike R190S. In both cases, other amino acids are replaced by l-serine. As explained for the mutation variant B.1.1.7, the spike protein has a copy number of 120. Changes in these structural proteins can be measurable and influence the stoichiometric coefficient stronger than mutations in non-structural proteins.

The mutation variant B.1.617 does not fit in this pattern. As the variants B.1.315 and B.1.427/429, its stoichiometric coefficients for ADP and ATP are increased, but not as much. Variant B.1.617 has a similar total viral molar mass as B.1.1.7. However, the amount of the nucleotides adenine and uridine is more similar to the variants B.1.315 and B.1.427/429. Having similarities with both pattern groups, variant B.1.617 does fit in neither of the groups. Variant B.1.617 needs less l-isoleucine compared to the wildtype.

#### 3.2.2. Analysis of the Effects of Single Gene Deletions

After highlighting the differences in the stoichiometric coefficients for the different mutation variants, we tested the robustness of our previously identified potential antiviral targets [22]. To do so, we repeated the single-gene-deletion experiments for every mutation variant. Our analysis revealed that in all mutation variants, the guanylate kinase 1 (GK1) is a robust potential antiviral target.

### 3.3. Lipids as Part of the Viral Biomass Objective Function

The transmembrane domain of the envelope (E) protein is located in lipid bilayers mimicking the ERGIC membrane [21]. Ref. [20] described this ERGIC membrane [20] in 1994. The four phospholipids, phosphatidylcholine, phosphatidylethanolamine, phosphatidylinositol, and phosphatidylserine, were observed in the ERGIC while sphingomyelin and cardiolipin were not present [20]. Ref. [21] use an ERGIC-mimetic consisting of the four described phospholipids and cholesterol to investigate the E-protein’s transmembrane domain [21]. The five lipids are also participating in the macrophage’s maintenance function. Thus, their role and influence on the VBOF and antiviral targets were examined.

As the actual amount of lipids in the SARS-CoV-2 virion is not yet determined, we evaluated varying stoichiometric coefficients. In the first experiments, the individual lipids’ effect on the VBOF’s objective value was analyzed. The objective coefficients from the macrophage’s maintenance function varied between 0.001 for phosphatidylserine and 0.031 for phosphatidylcholine. Therefore, we first varied all lipids’ coefficients between 0 and 0.5 and subsequently used a multiplication coefficient between 0 and 10 to multiply the macrophage’s coefficients. Despite an up to 490-fold increase of the stoichiometric coefficient (for phosphatidylserine) compared to its initial value in the macrophage’s maintenance function, the VBOF’s objective value remained at 0.01886 mmol/(gDW · h). This was also the case when all five lipids were added to the VBOF  simultaneously.

**Figure 5:**
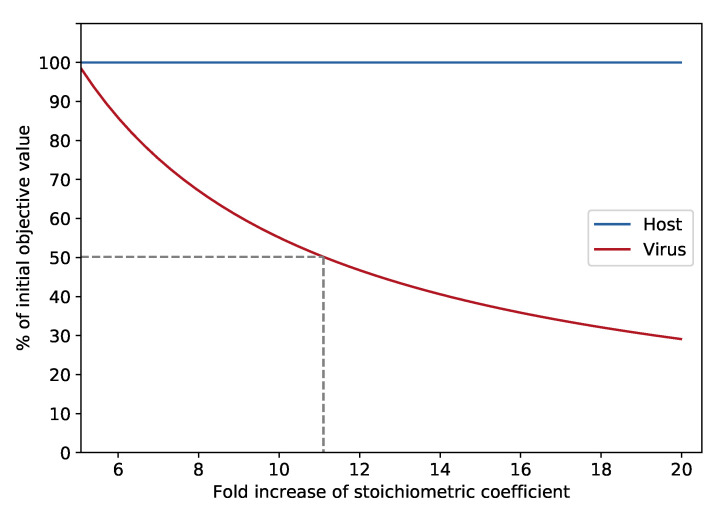
Influence of stoichiometric coefficient on reduction of VBOF during METS knock-out. With increasing factorization of phosphatidylcholine’s stoichiometric coefficient, the objective value of the VBOF’s optimization decreases during the knock-out of the methionine synthase (METS) reaction. The hosts growth maintenance stays at 100%. At an eleven-fold increase of the initial stoichiometric coefficient extracted from the host’s maintenance function results in a 50% decrease of the viral growth rate.

Knock-out experiments were conducted to identify additional potential antiviral targets. All lipids were included in the VBOF, and the coefficients were varied using a multiplication coefficient. At the five-fold increase of the initial stoichiometric coefficients, two novel reactions emerged as new potential antiviral targets: the methionine synthase (METS) and the 5,10-methylenetetrahydrofolate reductase (FADH_2_) (MTHFR). To identify, which lipids are responsible for the emergence of the novel antiviral target, we repeated the described analysis for every lipid individually, once using absolute stoichiometric coefficients ranging from 0 to 0.5 and once using the above-described multiplication coefficient ranging between 0 and 10. By this approach, we identified phosphatidylcholine to be the responsible lipid for the additional antiviral targets. When increasing the initial macrophage’s stoichiometric coefficient of phosphatidylcholine by at least 4.76, the two enzymes emerge as potential antiviral targets. At a five-fold increase of phosphatidylcholine and the knock-out of either the methionine synthase or the 5,10-methylenetetrahydrofolate reductase (FADH_2_), the viral growth can be inhibited by approximately 1.5%. With increasing amounts of phosphatidylcholine in the VBOF, the knock-out influence of the two reactions on the viral growth increases, as seen in Figure 5: at an eleven-fold increase of phosphatidylcholine, the viral growth rate is decreased by approximately 50%. A twenty-fold increase of phosphatidylcholine inhibits the viral growth even to 30% of its initial growth rate.

It needs to be highlighted that the guanylate kinase 1 (GK1) was a potential antiviral target during all conducted in silico experiments evaluating the lipids’ effect on potential targets.

## 4. Discussion

This study presents an updated viral biomass objective function (VBOF) for the novel coronavirus SARS-CoV-2 based on the latest information of its structural proteins. This VBOF was integrated into an already validated model of human alveolar macrophages [23].

The tissue tropism of SARS-CoV-2 comprises most cell types expressing the entry receptor ACE-2, mainly including cell types of the lung, liver, stomach, ileum, kidney, and colon [39,40]. Although SARS-CoV-2 enters the host via the airways, the expression of ACE-2 is comparably low, highlighting the role of possible co-receptors [40]. Nonetheless, human alveolar type 2 cells robustly express ACE-2, while alveolar macrophages possibly express low levels of the entry receptor [40]. It is known that different coronaviruses infect macrophages, such as the human coronavirus strain 229E [41], the Middle East Respiratory Syndrome (MERS) coronavirus [42], and the SARS coronavirus [43]. Also, the novel coronavirus SARS-CoV-2 is reported to infect alveolar macrophages [44]. However, other in vitro studies suggest that challenging alveolar macrophages with SARS-CoV-2 does not lead to a productive infection [45]. However, even without productive infection, alveolar macrophages could serve as Trojan horses, which enable viral anchoring within pulmonary parenchyma [39]. Ref. [45] demonstrate that the tissue-resident alveolar macrophages play a crucial role in SARS-CoV-2 immune evasion [44,45] and are hypothesized to support viral pathogenesis [39]. Disabling viral replication in human alveolar macrophages might be an early way of intervention and prevention of the virus’s further spread.

We corrected the copy number of structural proteins and the stoichiometric coefficients in the viral biomass objective function (VBOF). The amount of the spike (S) and nucleocapsid (N) proteins were derived from studies on SARS-CoV-2 [14,15,32]. The copy number of the envelope (E) protein is derived from the human coronavirus and the transmissible gastroenteritis virus [29]. Numbers for SARS-CoV-2 are currently not available. Same accounts for the copy number of membrane (M) proteins, where information is only available for SARS-CoV-1 [34]. Especially for the M proteins, a range of potential copy numbers exists, as the ratio of M and N proteins ranges from 3M:1N to 1M:1N [34]. With the N protein’s copy number of 456, the M protein’s copy number ranges from 456 to 1368. As soon as additional information on the copy numbers of the E and M protein is available for SARS-CoV-2, the stoichiometric coefficients can be refined further.

However, the current refinement still confirmed the guanylate kinase 1 (GK1) as a potential antiviral target. Even for the investigated mutations, the guanylate kinase seems to be a robust target in human alveolar macrophages to interrupt SARS-CoV-2 replication. Ref. [46] conduct a similar study with the human reconstruction RECON2.2 [47] containing a lung biomass objective function and a viral biomass objective function [46]. They also report the guanylate kinase as a potential target for antiviral therapies [46]. In our previous study, we suggested potential drugs that could be repurposed to fight this SARS-CoV-2 pandemic. Amongst these drugs were cidofovir, brincidofovir, and favipiravir [22]. A virtual screening method identified cidofovir as a potentially effective therapeutic against SARS-CoV-2 [48]. A molecular docking study suggests the repurposing of brincidofovir against SARS-CoV-2 [49]. For favipiravir, several clinical trials are listed in the ClinicalTrials database hosted by the U.S. National Library of Medicine [50], running in several countries, including Italy (NCT04336904), Turkey (NCT04474457), and the United States (NCT04358549). However, these therapeutics are only analogs and do not directly inhibit the guanylate kinase. No direct inhibitor of the guanylate kinase is tested for its antiviral effect on SARS-CoV-2 infections at the time of writing. As the guanylate kinase is a robust target for all currently occurring mutation variants, further investigations could be of high interest to fight this pandemic.

Besides the guanylate kinase, additional potential antiviral targets were identified using the host-derived enforcement analysis. These antiviral targets are located in the pentose phosphate pathway, the purine, and the pyrimidine metabolism. It is shown that the pentose phosphate pathway is remarkably deregulated during SARS-CoV-2 replication, which shows potential implications for antiviral therapies [51]. The purine biosynthesis pathway is enhanced upon SARS-CoV-2 infection to support the *de novo* synthesis of purines [52]. First in vitro experiments show that the FDA-approved inhibitor of purine biosynthesis methotrexate potently inhibits viral replication [53,54], protein synthesis, and release [53]. The pyrimidine metabolism is also reported as a potential antiviral target, especially the dihydroorotate dehydrogenase. Its inhibition by, for example, brequinar or leflunomide is already demonstrated to have antiviral activity against other viruses [55,56,57], such as rotavirus [58] and Ebola virus [59]. The dihydroorotate dehydrogenase inhibitor PTC299 is shown to arrest SARS-CoV-2 replication in vitro [60]. The dihydroorotate dehydrogenase inhibitors S312 and S416 are validated to have high antiviral efficacy in vivo [61]. To conclude, our identified antiviral targets are currently under discussion in the scientific community, and for some, the influence and relevance for viral replication are confirmed.

Analyses of the documented mutations revealed that virus variant B.1.617 needs less l-isoleucine compared to the wildtype because of a mutation in the membrane protein, M I82T, where l-isoleucine is substituted by l-threonine. As the membrane protein has a copy number of 1000, its replacement could influence the stoichiometric coefficient of the replaced amino acid. Same accounts for the mutation N D402H in the nucleocapsid protein, where l-aspartate is replaced by l-histidine, which might explain the decreased stoichiometric coefficient for l-aspartate. Changes in these structural proteins can be measurable and influence the stoichiometric coefficient stronger than mutations in non-structural proteins.

Alongside the mutation variants that could complicate the fight against SARS-CoV-2 with vaccines, the S protein’s glycosylation could impact antibodies’ ability to bind to a pathogenic S glycoprotein by shielding its surface [62,63]. Currently, this glycosylation process is not reflected in the VBOF or the model. As soon as more information about the glycosylation is available that can be used to determine a range or precise stoichiometric coefficients, the glycosylation of the spike protein can be incorporated into the model simulations.

The inclusion of lipids in the VBOF opens new perspectives for potential antiviral targets. It is shown that virus infections can dramatically impact on lipid metabolism [64,65,66,67]. Upon rhinovirus infection multiple lipid pathways are altered, and changes in phospholipids, lysophospholipids, fatty acids, and inositol phospholipids are observed [66]. For the human coronavirus 229E (hCoV-229E), the host cell lipid response upon infection was comprehensively characterized. Glycerophospholipids and fatty acids were significantly elevated. Lysophosphatidylcholine, which is hydrolyzed from phosphatidylcholine, was significantly elevated and accounted for approximately 60% of all identified lipids with significant elevation [68]. Our study also highlighted phosphatidylcholine as an essential lipid upon SARS-CoV-2 infection, confirming the findings from [68] for hCoV-229E. As metabolic alterations harbor potential antiviral targets, regulating or targeting the lipid metabolism is suggested and discussed [64,66,68]. We identified two novel potential antiviral targets connected with lipid metabolism: the methionine synthase and the 5,10-methylenetetrahydrofolate reductase (FADH_2_). S-adenosyl-l-methionine is a pivotal methyl donor in the synthesis of phosphatidylcholine [69,70]. Thus, the synthesis of l-methionine by the 5,10-methylenetetrahydrofolate reductase (FADH_2_) and methionine synthase seem to be an antiviral target to disrupt the synthesis of phosphatidylcholine. These novel insights could guide further laboratory experiments for investigating and validating the lipid’s role in SARS-CoV-2 infections.

This study confirmed the guanylate kinase 1 (GK1) as a robust antiviral target against SARS-CoV-2 and its arising mutation variants. With the refined copy numbers of structural proteins, the list of further potential antiviral targets was improved, and some targets are already under discussion or even under validation. The inclusion of the lipids into the VBOF opened new perspectives for additional metabolic targets to fight against this pandemic.

## Figures and Tables

**Table 1:** Stoichiometric coefficients of the five lipids in the macrophage’s maintenance function. The stoichiometric coefficients of the five lipids were extracted from the macrophage’s maintenance function. Additionally, the BiGG identifiers [28] of the lipids are given. These stoichiometric coefficients formed the starting point for evaluating the lipids’ influence on the viral biomass objective function (VBOF).

Lipid	BiGG ID	Coefficient
Phosphatidylcholine	pchol_hs_c	0.03152
Phosphatidylethanolamine	pe_hs_c	0.02110
Phosphatidylinositol	pail_hs_c	0.00374
Phosphatidylserine	ps_hs_c	0.00102
Cholesterol	chsterol_c	0.02093

**Table 2:** Copy number of structural proteins. The Copy number of structural proteins (Csp) was determined based on extensive literature research. Besides the reference and the copy number of structural proteins, the investigated organism is given as a source.

Protein	Name	Reference	Source	Csp
S	S	[15]	SARS-CoV-2	120
E	E	[29]	hCOV, TGEV	20
N	N	[14,32]	SARS-CoV-2	456
M	M	[34]	SARS-CoV-1	1000

## Data Availability

The genome-scale metabolic model of the human alveolar macrophage infected with SARS-CoV-2 is available in the BioModels Database [71] as an SBML Level 3 Version 1 file [72,73,74] with the flux balance constraints (fbc) extension package [75] within a COMBINE Archive OMEX file [76] under the accession number MODEL2003020001. A supplementary table in Microsoft Excel format is available as Supplementary Materials along with this article.

## Supplementary Materials

The following are available online at https://www.mdpi.com/article/10.3390/genes12060796/s1, Table S1: List of tested variants. The tested variants are listed together with their GISAID accession number and the observed mutations. The stoichiometric coefficients for the VBOF’s compounds in the different virus mutations are listed.

## Abbreviations

The following abbreviations are used in this manuscript:

ACE-2	angiotensin converting enzyme 2
ADP	adenosine diphosphate
ADSL1	adenylosuccinate lyase 1
ADSL2	adenylosuccinate lyase 2
ADSS	adenylosuccinate synthase
AICART	phosphoribosylaminoimidazolecarboxamide formyltransferase
AIRCr	Phosphoribosylaminoimidazole carboxylase
AMP	adenosine monophospate
ASPCTr	aspartate carbamoyltransferase
ATP	adenosine triphosphate
CBPS	carbamoyl-phosphate synthase
COMBINE	Computational Modeling in Biology Network
COVID-19	Coronavirus Disease 19
Csp	Copy number of structural proteins
DHORD9	dihydoorotic acid dehydrogenase
DHORTS	dihydroorotase
E	envelope
ERGIC	endoplasmic reticulum–Golgi intermediate compartment
FBA	flux balance analysis
fbc	flux balance constraints
FVA	flux variability analysis
GARFT	phosphoribosylglycinamide formyltransferase
GEM	genome-scale metabolic model
GISAID	Global Initiative on Sharing All Influenza Data
GK1	guanylate kinase 1
GLUPRT	glutamine phosphoribosyldiphosphate amidotransferase
hCOV	human coronavirus
hCoV-229E	human coronavirus 229E
ID	identifier
IMP	inosine monophosphate
IMPC	IMP cyclohydrolase
M	membrane
MERS	Middle East Respiratory Syndrome
Mv	viral molar mass
N	nucleocapsid
OMEX	Open Modeling EXchange format
OMPDC	orotidine-5’-phosphate decarboxylase
ORPT	orotate phosphoribosyltransferase
PPi	diphosphate
PRAGSr	phosphoribosylglycinamide synthase
PRAIS	phosphoribosylaminoimidazole synthase
PRASCS	phosphoribosylaminoimidazolesuccinocarboxamide synthase
PRFGS	phosphoribosylformylglycinamidine synthase
PRPPS	phosphoribosylpyrophosphate synthetase
RPI	ribose-5-phosphate isomerase
S	spike
SARS	Severe Acute Respiratory Syndrome
SARS-CoV-2	Severe Acute Respiratory Syndrome coronavirus type 2
SBML	Systems Biology Markup Language
TGEV	transmissible gastroenteritis virus
VBOF	viral biomass objective function
vRNP	viral ribonucleoprotein complexes
WT	wildtype

## Appendix A

**Table A1:** Reactions from the host-derived enforcement experiments. The reaction identifiers listed in Figure 2 are BiGG identifiers [28]. In this table, the BiGG reaction identifiers are given, together with the reaction name and the subsystem, they occur in.

Reaction-ID	Reaction Name	Subsystem
ADSL1	adenylosuccinate lyase 1	Purine metabolism
ADSL2	adenylosuccinate lyase 2	Purine metabolism
ADSS	adenylosuccinate synthase	Purine metabolism
AICART	phosphoribosylaminoimidazolecarboxamide formyltransferase	Purine metabolism
AIRCr	Phosphoribosylaminoimidazole carboxylase	Purine metabolism
ASPCTr	aspartate carbamoyltransferase	Pyrimidine metabolism
CBPS	carbamoyl-phosphate synthase	Pyrimidine metabolism
DHORD9	dihydoorotic acid dehydrogenase	Pyrimidine metabolism
DHORTS	dihydroorotase	Pyrimidine metabolism
GARFT	phosphoribosylglycinamide formyltransferase	Purine metabolism
GK1	guanylate kinase 1	Purine metabolism
GLUPRT	glutamine phosphoribosyldiphosphate amidotransferase	Purine metabolism
IMPC	IMP cyclohydrolase	Purine metabolism
OMPDC	orotidine-5’-phosphate decarboxylase	Pyrimidine metabolism
ORPT	orotate phosphoribosyltransferase	Pyrimidine metabolism
PRAGSr	phosphoribosylglycinamide synthase	Purine metabolism
PRAIS	phosphoribosylaminoimidazole synthase	Purine metabolism
PRASCS	phosphoribosylaminoimidazolesuccinocarboxamide synthase	Purine metabolism
PRFGS	phosphoribosylformylglycinamidine synthase	Purine metabolism
PRPPS	phosphoribosylpyrophosphate synthetase	Pentose phosphate pathway
RPI	ribose-5-phosphate isomerase	Pentose phosphate pathway
